# LEGO-CSM: a tool for functional characterization of proteins

**DOI:** 10.1093/bioinformatics/btad402

**Published:** 2023-06-29

**Authors:** Thanh Binh Nguyen, Alex G C de Sá, Carlos H M Rodrigues, Douglas E V Pires, David B Ascher

**Affiliations:** School of Chemistry and Molecular Biosciences, University of Queensland, Brisbane City, QLD 4072, Australia; Systems and Computational Biology, Bio21 Institute, University of Melbourne, Parkville, VIC 3052, Australia; Computational Biology and Clinical Informatics, Baker Heart and Diabetes Institute, Melbourne, VIC 3004, Australia; School of Chemistry and Molecular Biosciences, University of Queensland, Brisbane City, QLD 4072, Australia; Systems and Computational Biology, Bio21 Institute, University of Melbourne, Parkville, VIC 3052, Australia; Computational Biology and Clinical Informatics, Baker Heart and Diabetes Institute, Melbourne, VIC 3004, Australia; Baker Department of Cardiometabolic Health, University of Melbourne, Parkville, VIC 3010, Australia; School of Chemistry and Molecular Biosciences, University of Queensland, Brisbane City, QLD 4072, Australia; Systems and Computational Biology, Bio21 Institute, University of Melbourne, Parkville, VIC 3052, Australia; Computational Biology and Clinical Informatics, Baker Heart and Diabetes Institute, Melbourne, VIC 3004, Australia; Systems and Computational Biology, Bio21 Institute, University of Melbourne, Parkville, VIC 3052, Australia; Computational Biology and Clinical Informatics, Baker Heart and Diabetes Institute, Melbourne, VIC 3004, Australia; School of Computing and Information Systems, University of Melbourne, Parkville, VIC 3052, Australia; School of Chemistry and Molecular Biosciences, University of Queensland, Brisbane City, QLD 4072, Australia; Systems and Computational Biology, Bio21 Institute, University of Melbourne, Parkville, VIC 3052, Australia; Computational Biology and Clinical Informatics, Baker Heart and Diabetes Institute, Melbourne, VIC 3004, Australia; Baker Department of Cardiometabolic Health, University of Melbourne, Parkville, VIC 3010, Australia; School of Computing and Information Systems, University of Melbourne, Parkville, VIC 3052, Australia

## Abstract

**Motivation:**

With the development of sequencing techniques, the discovery of new proteins significantly exceeds the human capacity and resources for experimentally characterizing protein functions. Localization, EC numbers, and GO terms with the structure-based Cutoff Scanning Matrix (LEGO-CSM) is a comprehensive web-based resource that fills this gap by leveraging the well-established and robust graph-based signatures to supervised learning models using both protein sequence and structure information to accurately model protein function in terms of Subcellular Localization, Enzyme Commission (EC) numbers, and Gene Ontology (GO) terms.

**Results:**

We show our models perform as well as or better than alternative approaches, achieving area under the receiver operating characteristic curve of up to 0.93 for subcellular localization, up to 0.93 for EC, and up to 0.81 for GO terms on independent blind tests.

**Availability and implementation:**

LEGO-CSM’s web server is freely available at https://biosig.lab.uq.edu.au/lego_csm. In addition, all datasets used to train and test LEGO-CSM’s models can be downloaded at https://biosig.lab.uq.edu.au/lego_csm/data.

## 1 Introduction

Proteins are versatile macromolecules that orchestrate most biological processes (BPs). Advances in next-generation sequencing techniques generated an explosion in the number of novel sequencing data available with potential for biomedical and pharmaceutical applications, including a better understanding of links between disease and evolutionary processes (Kissa *et al.* 2015, Zeng *et al.* 2016, Zhang *et al.* 2019, Uthayopas *et al.* 2021), development of new drugs (Xuan *et al.* 2019, Barabasi *et al.* 2011), and many other domains (Zheng and Wang 2008, Mi *et al.* 2013, Radivojac *et al.* 2013; Jiang *et al.* 2016, Zhou *et al.* 2019). However, experimental functional characterization of new proteins is a costly, complex, and time-consuming endeavour. As a result, as of June 2022, only just 0.25% of all available sequences in UniProt (2021) have been manually annotated and reviewed.

To overcome this challenge, a range of computational tools has been developed to efficiently annotate specific aspects of protein functions, including subcellular localization (Almagro Armenteros *et al.* 2017), enzymatic activity [using the enzyme commission (EC) number], and broad biological and molecular functions (MFs) [using the Gene Ontology (GO) terms] (Kulmanov and Hoehndorf 2020). Most computational approaches, however, have focused on only one or two functional areas, and have primarily relied on sequence-derived information. Although there are methods that have used structure information for these tasks, most focus solely on high-quality experimentally determined structures (Gao *et al.* 2019, Gligorijevic *et al.* 2021), which are limited to 76 800 entries available in PDB as of February 2023. Nevertheless, recent breakthroughs in the prediction of protein structures, led by AlphaFold2 (Jumper *et al.* 2021) and RoseTTAFold (Baek *et al.* 2021), have revolutionized the field of structural biology and opened the possibility of exploring the entire proteome (Akdel *et al.* 2021).

Here, we propose combining advances in both functional and structural modelling to understand protein functions; particularly subcellular localization, enzymatic activity, and GO term classification, more accurately and comprehensively with LEGO-CSM: Localization, EC numbers, and GO terms with the structure-based Cutoff Scanning Matrix algorithm. LEGO-CSM outperformed or performed comparable to state-of-the-art methods in each area, providing a unified platform to enable rapid assessment of all three protein functional domains. Our platform is publicly available as a user-friendly web server and API to facilitate large-scale processing.

## 2 Methods

**Figure 1.**
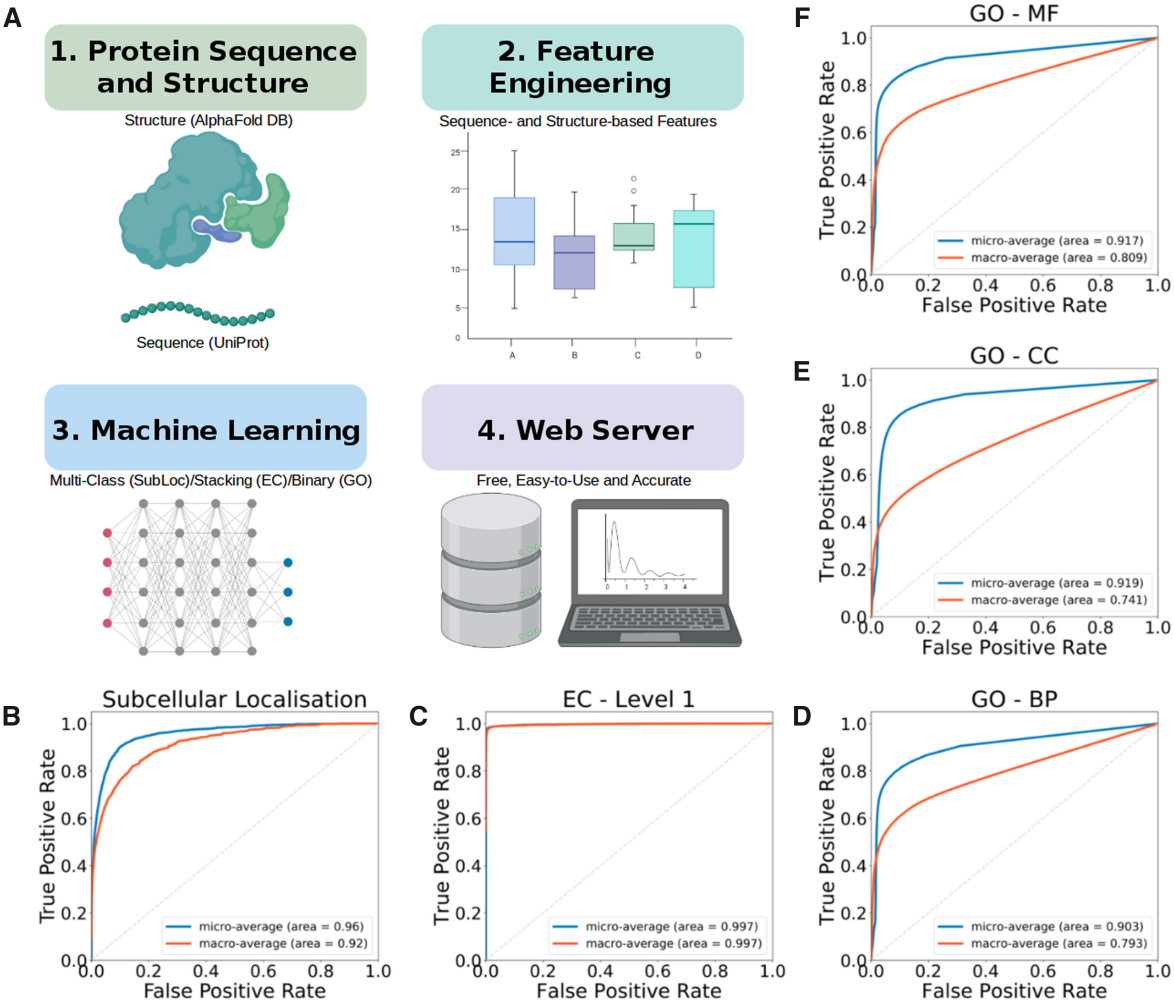
LEGO-CSM workflow and predictive performance. (A) LEGO-CSM methodological workflow, composed of four main stages, including data collection and curation, feature engineering, development of predictive models using supervised machine learning, and development of a web server. (B–F) The micro- and macro-averaged ROC AUC performances of the five predictive models developed on blind test data (subcellular localization, EC number at level 1, GO BP, GO CC, and GO MF, respectively).

Figure 1A depicts LEGO-CSM’s workflow comprised of four main steps: (1) dataset acquisition and curation of protein sequences and structures, (2) feature engineering, (3) development and validation of predictive models via supervised learning, and (4) deployment of best-performing models in a friendly web-based interface.

Sequence-based features were extracted from the amino acid composition, physicochemical properties, disorder propensity scores, and secondary structure content (Supplementary Data). Structure-based features comprised (1) graph-based signatures—a method that represents proteins as graphs to model geometry and physicochemical properties using the CSM algorithm (Pires *et al.* 2014), (2) residue depth, (3) solvent accessible surface area, (4) secondary structure distribution, and (5) torsion angles.

Detailed information about datasets used for each predictive task (Supplementary Fig. S1 and Supplementary Tables S1 and S2) is available as Supplementary Data, as well as, specifications on machine learning workflows, feature selection and web server development. In addition, the best predictive models for each functional annotation type (subcellular localization, EC, and GO) were benchmarked with state-of-the-art methods and made available via our web server and API (Supplementary Figs S2 and S3).

## 3 Results

### 3.1 Performance of LEGO-CSM models

Supplementary Table S3 summarizes LEGO-CSM’s performance on cross-validation (CV) and independent blind tests. The proposed subcellular localization model for 10 different cellular compartments achieved an average area under the receiver operating characteristic curve (ROC AUC) of up to 0.970 (Supplementary Fig. S4) and an accuracy of 0.757 (Supplementary Table S4) under 10-fold CV. Performance measures are reported as weighted averages given the imbalanced nature of the classes in the dataset (Supplementary Fig. S1 and Supplementary Table S5). The model’s predictive performance on the independent blind test was consistent with the CV on training (Fig. 1B and Supplementary Table S4), demonstrating its robustness and general capabilities.

At the ‘deepest’ EC level—i.e. with the largest number of classes and data sparsity—under CV, LEGO-CSM using sequence information achieved F1-score and MCC of 0.951 and 0.804, respectively, while on the blind tests, the corresponding values were 0.908 and 0.730, respectively (Supplementary Tables S6–S9).

For predicting GO terms, we built a binary classifier for each GO term and achieved under CV ROC AUCs of 0.731, 0.742, and 0.805 for BP, cellular component (CC), and MF, respectively, using only sequence information (Supplementary Tables S3 and S10). A compatible performance on independent blind test sets was also obtained (ROC AUCs of 0.792, 0.741, and 0.809 for BP, CC, and MF, respectively), indicating good generalization capabilities.

Structural information was incorporated into the feature set to build predictive models for EC and GO terms and achieved compatible performance (Supplementary Tables S3 and S6), even having a lower amount of structural data, showing an overall great generalization.

### 3.2 Comparison between LEGO-CSM and alternative methods

We compared LEGO-CSM subcellular localization with three alternative methods (Supplementary Table S4) using a blind test set. LEGO-CSM has shown better (Plant-mSubP) or comparable (MuLocDeep and DeepLoc) predictive performances in terms of Gorodkin (2004) and accuracy measures.

Our model for EC prediction was compared with four state-of-the-art methods currently available (Supplementary Table S6): ECPred, Benz WS, DeepEC (Ryu *et al.* 2019), and DeepFRI (Gligorijevic *et al.* 2021). From level 0 to level 3, LEGO-CSM presented either comparable (ECPred) or better (Benz WS, DeepFRI, and DeepEC) performance, while at level 4 our models, using sequence-only information and adding 3D structure data outperformed all four methods, highlighting the importance of using structural information to achieve robust predictive performances even with an unbalanced training set. Although DeepFRI uses both sequence and structure information, its performance is worse than LEGO-CSM.

Finally, we compared our models for predicting GO terms to DeepGOPlus, NetGO 2.0 (Yao *et al.* 2021), and DeepFRI. Results showed that LEGO-CSM outperformed or performed as well as DeepGOPlus, NetGO 2.0, and DeepFRI in terms of ROC AUC and balanced accuracy measures across all GO subontologies (BP, CC, and MF) (Supplementary Tables S10 and S11) while LEGO-CSM showed a similar or slightly lower performance to those alternative methods in the area under the precision–recall curve and *F*_max_ (Supplementary Tables S12 and S13). Nevertheless, the performance of the top 10 imbalance and balance classes showed the ability of LEGO-CSM to predict even low frequency labels (Supplementary Table S14).

## 4 Discussion

In this study, we introduced LEGO-CSM, a novel web-based platform developed to provide a unified resource to integrate three protein function components: (1) subcellular localization, (2) enzymatic function, and (3) gene ontology terms, which leverage both protein sequence and structure information. To ensure its effectiveness, we compared LEGO-CSM to alternative methods and demonstrated our models performed as well as or better than state-of-the-art approaches. LEGO-CSM is freely available as an easy-to-use web-based platform at http://biosig.lab.uq.edu.au/lego_csm/. LEGO-CSM provides an invaluable resource and tool to explore protein function characterization, guiding experimental validation and supporting the development of biomedical and pharmaceutical applications.

## Supplementary Material

btad402_Supplementary_Data

## Data Availability

The data underlying this article are available at: http://biosig.lab.uq.edu.au/lego_csm/.
