# Supplementary material for: LEGO-CSM: a tool for functional characterization of proteins
Source: Bioinformatics. 2023 Jun 29;39(7):btad402. doi: 10.1093/bioinformatics/btad402 (PMC10329489; doi:10.1093/bioinformatics/btad402)
Supplement: btad402_Supplementary_Data [file btad402_supplementary_data.docx]

**Supplementary Materials**

**LEGO-CSM: a tool for functional characterisation of proteins**

Thanh Binh Nguyen^1,2,3,#^, Alex G.C. de Sá^1,2,3,4,#^, Carlos H. M. Rodrigues^1,2,3,#^,

Douglas E. V. Pires^2,3,5,*^, David B. Ascher^1,2,3,4,*^

^1^ School of Chemistry and Molecular Biosciences, University of Queensland, Brisbane City, Queensland, 4072, Australia

^2^ Systems and Computational Biology, Bio21 Institute, University of Melbourne, Parkville, Victoria, 3052, Australia

^3^ Computational Biology and Clinical Informatics, Baker Heart and Diabetes Institute, Melbourne, Victoria, 3004, Australia

^4^ Baker Department of Cardiometabolic Health, University of Melbourne, Parkville, Victoria, 3010, Australia

^5^ School of Computing and Information Systems, University of Melbourne, Parkville, Victoria, 3052, Australia

**^#^** These authors equally contributed to the paper.

*To whom correspondence should be addressed

Contact: [d.ascher@uq.edu.au](mailto:d.ascher@uq.edu.au), [douglas.pires@unimelb.edu.au](mailto:douglas.pires@unimelb.edu.au).

**Materials and methods**

**Datasets**

*Subcellular localisation datasets*

A subcellular localisation model was trained and tested on datasets comprising 10 different cellular compartments, namely: cell membrane, cytoplasm, endoplasmic reticulum, Golgi apparatus, lysosome/vacuole, mitochondrion, nucleus, peroxisome, plastid and extracellular. The training set is composed of 11,231 proteins, with an independent blind test including 2,773 proteins obtained from (Almagro Armenteros, et al., 2017). The distribution of proteins per label for each data set is shown in Figure S1, highlighting a considerable class imbalance, however consistent proportions per class between data sets. In both training and blind test sets, the most abundant labels were proteins found in the nucleus, while the least abundant proteins were in the peroxisome.

*EC number datasets*

A sequence-based EC number prediction model was trained and assessed on a manually annotated dataset comprising 137,015 enzymes having 90% sequence identity cutoff obtained from UniProt in Sep 2021 (Dalkiran, et al., 2018), belonging to six main reaction classes namely: oxidoreductases, transferases, hydrolases, lyases, isomerases and ligases, also including a control group of 34,254 non-enzymes. An independent blind set (Dalkiran, et al., 2018) with a cutoff of 50% sequence identity from the training set comprising 5,516 enzymes and 1,810 non-enzymes was also employed. The total number of labels at the last level of the EC hierarchy was 859. Table S1 depicts the label distribution for the first EC level, also highlighting a significant class imbalance. A detailed description of EC number terms can be found at <https://biosig.lab.uq.edu.au/lego_csm/data>. Another predictive model was trained and evaluated on a subset of proteins for which structural information was available. Only high-quality AlphaFold2 structures were considered, with a confidence score threshold higher or equal to 50 pLDDT (Mariani, et al., 2013). The training dataset contained 20,673 enzymes and 34,250 non-enzymes. A non-redundant blind set was also considered, including 1,500 enzymes and 1,518 non-enzymes (Table S1 shows the class distribution for level 1 of the hierarchy).

*GO terms datasets*

Sequence-based GO predictive models were developed using a dataset comprising 45,873 proteins labelled with biological processes (BP), 45,250 proteins labelled with molecular function (MF) and 45,164 with cellular components (CC) (Table S2) (Sureyya Rifaioglu, et al., 2019). Three predictive models were developed, one for each label type. Generalisation capabilities were assessed using an independent blind set, including 15,301 proteins labelled with BP, 10,785 with MF and 11,216 with CC. Structure-based models were trained on a subset of proteins for which high-quality AlphaFold2 models were available (following the same criteria as for EC models), leading to 39,699 proteins for BP, 38,257 for MF and 41,403 for CC classes (with 13,510, 9,268 and 10,552 proteins utilised as blind test sets for the BP, MF and CC models, respectively).

**Feature generation**

*Sequence-based features* were generated using 4 main approaches: (1) iLearn (Chen, et al., 2021; Chen, et al., 2018; Chen, et al., 2020) was used to extract information on amino acid composition, physicochemical properties and AAINDEX scores (Kawashima and Kanehisa, 2000), (2) disorder propensity scores were derived using IUPred2 (Meszaros, et al., 2018); (3) secondary structure content was calculated using S4Pred (Moffat and Jones, 2021), and (4) a set of physicochemical properties and indexes were extract from the Peptides R package, including scores from BLOSUM matrices and hydrophobicity index.

*Structure-based features* include:

(1) Graph-based signatures: represent proteins as graphs to model geometry and physicochemical properties using the CSM algorithm (Pires, et al., 2014). These have been applied successfully in a range of scenarios, including predicting protein structural classes (Pires, et al., 2011), modelling mutation effects (Myung, et al., 2020; Myung, et al., 2020; Nguyen, et al., 2021; Pires and Ascher, 2016; Pires, et al., 2016; Pires and Ascher, 2017; Pires, et al., 2020; Rodrigues, et al., 2018; Rodrigues, et al., 2019; Rodrigues, et al., 2021; Rodrigues, et al., 2021), predicting drug resistance (Karmakar, et al., 2018; Karmakar, et al., 2019; Karmakar, et al., 2020; Portelli, et al., 2020; Portelli, et al., 2018; Vedithi, et al., 2018; Vedithi, et al., 2020), identifying epitope regions (da Silva, et al., 2022) and as docking scoring functions (Myung, et al., 2021; Nguyen, et al., 2022; Pires and Ascher, 2016). Molecular graphs are generated to represent each protein, with atoms modelled as nodes and interactions as edges. Nodes are labelled based on seven atom types, namely: aromatic, hydrophobic, negative, positive, hydrogen bond donor, hydrogen bond acceptor, and neutral (Pires, et al., 2014). The cumulative distribution of distances between atom types in the graphs is extracted and represented as a feature vector.

(2) Complementary structural features: residue depth, solvent accessible surface area, secondary structure distribution, and torsion angles were calculated using Biopython package (Hamelryck and Manderick, 2003) for each residue, and average values were employed.

Features with low discrimination power (low variance) were removed using the variance threshold method available on the scikit-learn Python package (Pedregosa, et al., 2011). Features were removed if they had a variance lower than 1.0. In addition, groups of features were assessed within the predictive models based on their performance on the classification tasks and prediction scores were also incorporated (Almagro Armenteros, et al., 2017; Dalkiran, et al., 2018; Kulmanov and Hoehndorf, 2020).

**Machine learning approach**

*Subcellular Localisation Model*

For predicting subcellular localisation, LEGO-CSM employs a multi-class classification approach to predict 10 different cellular compartments. A range of classification algorithms was tested, including Adaptive Boosting, Gradient Boosting, Extreme Gradient Boosting (XGBOOST), Random Forest and Extremely Randomized Trees. XGBOOST obtained the best predictive performance in terms of Matthew’s correlation coefficient (MCC) and ROC AUC over a 10-fold cross-validation procedure on the training set (validation procedure consistent with alternative methods used in performance comparisons). We employed MCC and ROC AUC to guarantee we have at least one measure (in this case, MCC) that could deal with high levels of class imbalance.

*Enzyme Commission (EC) Model*

LEGO-CSM encompasses a stacking-based classification approach (Wolpert, 1992) for building models for predicting the four levels of the EC hierarchy. In this approach, the predictions of one EC level serve as features for the next level. Hence, one level aggregates information for the subsequent one, with level 0 denoting enzymes and non-enzymes. The same set of binary classification algorithms was assessed with XGBOOST presenting the best results for level 0 under 5-fold cross-validation (validation procedure consistent with previous works) based on MCC and F1-measure. For the remaining levels, a multi-label classification approach was employed (Herrera, et al., 2016; Zhang and Zhou, 2014). Binary Relevance and Classifier Chains multi-label methods were assessed and combined with the five classification algorithms used at level 0. The best-performing multi-label classification strategy was considering the Binary Relevance method with an XGBOOST classifier. The XGBOOST predictors employed 300 models in their ensemble.

*Gene Ontology (GO) Models*

While predicting Gene Ontology (GO) terms, LEGO-CSM includes one binary classification model per GO term. This decision resulted in 2,662 models considering all three GO categories, *i.e.*, 1,895 models for BP, 422 models for MF and 345 models for CC. Given the high level of complexity in predicting GO terms, we assessed three classification algorithms, namely XGBOOST, Random Forest and Extremely Randomized Trees. Feature selection was performed using these algorithms to retrieve the 10 most important sequence-based and structure-based features for each GO term predictive model, and threshold calibration was utilised on the classification probability scores to assure robust predictive performances. Overall, the probability scores (or confidence scores) mean the certainty of the model to classify that particular GO term in being associated with a particular protein. The higher the value is, the more reliable the prediction. However, given high levels of class imbalance, Random Forest using a classification threshold of 0.1 yielded the best results based on ROC AUC and Balanced Accuracy (BACC) across a 10-fold cross-validation procedure for all three GO categories, a validation procedure consistent with previous works.

*Performance metrics and validation procedures*

The main performance metrics used to assess LEGO-CSM include MCC, ROC AUC, area under the precision-recall (AUPRC), F1-score, F_max_ and BACC. MCC is a robust classification metric, particularly relevant in scenarios where a large class imbalance is present. MCC considers false and true positive and negative predictions to yield a balanced predictive coefficient, which can evaluate a classifier even when classes have very different proportions. MCC is a correlation-based measure that varies from –1 to +1, where +1 indicates a perfect positive correlation, 0 denotes a random classifier, and –1 is a perfect inverse correlation. ROC AUC, in turn, is a robust classification measure based on the ROC curve, which is plotted with the true positive rate (TPR) and false negative rate (FNR) by considering several threshold levels. The ROC AUC score is measured by taking the area under the curve derived by TPR and FNR. ROC AUC score is defined in the interval of 0.0 to 1.0, where a ROC AUC of 1.0 indicates an optimal classifier and a ROC AUC of 0.5 means the model has the same performance as a random binary model.

F1-score, F_max,_ AUPRC and BACC are measures commonly used to evaluate models on imbalanced datasets. F1-score is an average metric based on a harmonic mean between recall (true positive rate) and precision across all samples. F_max_ takes the *arg max* of the F1-score across all samples. Therefore, F_max_ is a local-based measure, not considering the overall global prediction of the model. Differently, the AUPRC summarises the precision-recall curve, which performs a tradeoff between precision and recall considering distinct classification probability thresholds. Finally, BACC yields non-inflated estimates while evaluating the predictions by equivalently macro-averaging the recall scores in each class.

*Benchmarking*

We compared our subcellular localisation model with several other localisation methods, namely CELLO (Yu, et al., 2006), DeepLoc, Plant-mSubP (Sahu, et al., 2020), and MuLocDeep (Jiang, et al., 2021). Our enzyme commission (EC) model was compared with EcPred, Benz WS (Baldazzi, et al., 2021), DeepEC (Ryu, et al., 2019) and DeepFRI (Gligorijevic, et al., 2021) and the Gene Ontology (GO) Models with DeepGOPlus (Kulmanov and Hoehndorf, 2020), NetGO 2.0 (Yao, et al., 2021) and DeepFRI.

**Web server**

*Input*

LEGO-CSM allows users to characterise a range of protein functions based on sequence and structural information. Users are required to provide a protein sequence in FASTA format and have the option to provide a protein structure by uploading a file in PDB format or providing a valid PDB accession code (Figure S2). Alternatively, users may query structures available on the AlphaFold2 database using protein name or UniProt accession code. Examples and format descriptions are available both on the submission page and the help page via the top navigation menu.

*Output*

Predictions are presented on the output page in 4 sections (Figure S3): (1) the predicted EC number is shown at the top of the page, including a description for the predicted class, subclasses and a reference to the BRENDA database of enzymes(Schomburg, et al., 2017)￼. This section also includes the predicted GO terms with their description, confidence score, ontology and a reference to AmiGO(Carbon, et al., 2009)￼; (2) predicted subcellular localisation is displayed via an interactive viewer of the cell using the SwissBioPics web (Le Mercier, et al., 2022) component developed by the Swiss-Prot group; (3) if a protein structure was provided during job submission, this section will show an interactive 3D viewer built using the NGLviewer (Rose, et al., 2018); (4) lastly, the details of the input protein sequence are shown at the bottom of the page, including amino acid composition, molecular weight, isoelectric point, molar extinction coefficient and ultraviolet absorbance.

***Processing Time***

The overall average processing time for LEGO-CSM jobs is around 20 minutes for proteins up to 1,600 amino acids long. For much larger protein sequences (2,772 amino acids), prediction time had average closer to 30 minutes. Figure S5 summarises the processing time of LEGO-CSM for different size proteins. For each protein size, 10 repetitions were executed.

*Processing infrastructure*

LEGO-CSM is implemented as a user-friendly and freely available web server. The server front end was built using the Materialize framework version 1.0.0, and the back end was developed using Python via the Flask framework (version 1.0.2). The web server is hosted on a Linux Server running Apache2.

**RESULTS AND DISCUSSION**

**Performance of LEGO-CSM models**

*Localisation prediction*

It is worth noting that given the high level of class imbalance in subcellular localisation data (Figure S1), all performance measures were proportionally averaged across the different compartment class sizes. Dealing with an unbalanced dataset is a well-known challenge for machine learning methods, which is observed here by the variable true positive ratio (recall) based on class abundance (Table S5).

*EC prediction*

In LEGO-CSM, we build a multi-label predictive model for each level of the EC hierarchy, where the last level is the most challenging one given the larger number of classes and their respective sparsity (including a total of 859 classes with an average of 160 proteins per class). For predicting enzymatic *versus* non-enzymatic classes (level 0), LEGO-CSM was able to obtain excellent classification performance, presenting an F1-score and MCC of up to 0.986 and 0.932, respectively, on a 5-fold cross-validation procedure over the training set (Table S6). Similarly, in a blind test set evaluation, LEGO-CSM showed at level 0 an F1-score and MCC of up to 0.982 and 0.931, demonstrating good generalisation capabilities. In the same direction, while distinguishing the six main EC classes at level 1, LEGO-CSM achieved scores that were higher than 0.909 for both F1-score and MCC measures on 5-fold cross-validation over the training set and blind test set, considering either sequence-based data only (Table S7) or sequence and structure information (Table S8). Figure 1C also denotes the macro- and micro-average top ROC AUC performances at level 1 on the blind test set, which shows that LEGO-CSM can generalise when facing novel protein data. At the intermediate levels (levels 2 and 3), good predictive performances were demonstrated by LEGO-CSM in terms of F1-score and MCC (Table S6), even with a limited amount of data to train the models. At level 4, using solely sequence information, on 5-fold cross-validation, our predictive model achieved an F1-score and MCC of 0.951 and 0.804, respectively (Table S6), which was consistent with the performance obtained on a non-redundant blind set (F1-score and MCC of 0.908 and 0.730, respectively), increasing confidence in the model's predictive abilities and reducing risks of overfitting. LEGO-CSM achieved equivalent performance when considering both sequence and structure information (F1-score and MCC of 0.844 and 0.779, respectively, on 5-fold cross-validation and 0.864 and 0.774 on blind tests). The performance of the top 10 imbalance and balance classes showed the ability of LEGO-CSM to predict even low-frequency labels (Table S9).

*GO prediction*

While GO does not have a full hierarchical structure, terms are organised into three sub-ontologies (BP, CC and MF), and proteins can be assigned to multiple GO terms at the same time. We trained individual predictive models for each of the GO terms in the sub-ontologies, as binary classifiers on both sequence information alone (main text), and with additional structural information. When additional structural information was incorporated into the feature set, a slight increase in the predictive performance on cross-validation was observed (ROC AUC performance of BP, CC, MF of 0.747, 0.748, and 0.805, respectively), with consistent predictive performances on the blind tests (ROC AUC performance of BP, CC, MF of 0.786, 0.728, and 0.807, respectively). Performances are also depicted in Figures 1D, 1E and 1F, where macro- and micro-average ROC AUC plots across the blind test sets are shown for BP, CC and MF sub-ontologies, respectively.

It is worth noting that these good predictive results for GO models were reached even facing considerable data imbalance, which is demonstrated in Table S14. In this table, the performance of the top 5 imbalanced and balanced classes shows the ability of LEGO-CSM to predict low-frequency labelled data (Table S14). In future work, we intend to deal with rare labels by employing cost-sensitive learning approaches.

**Figures**


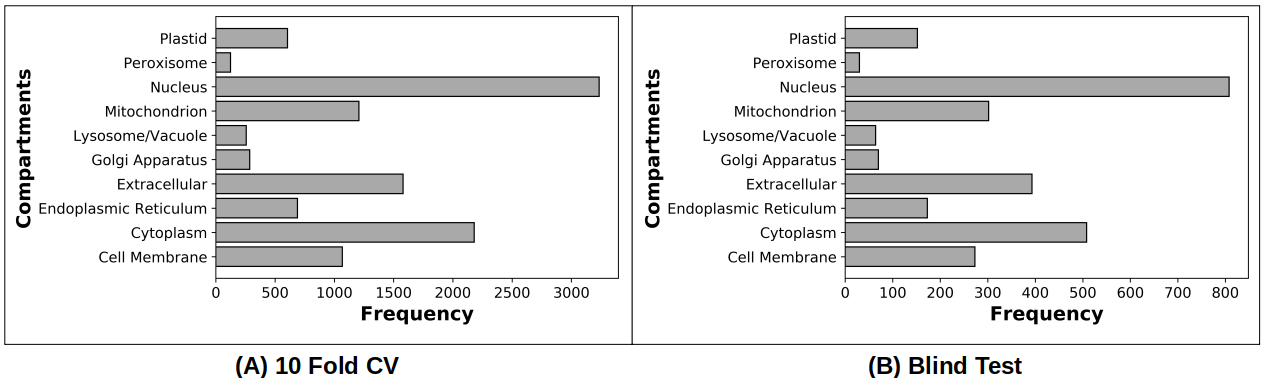
**Figure S1.** Frequency of compartment classes for subcellular localisation data in LEGO-CSM. Frequencies are shown across the training set used for 10-fold cross-validation (A) and blind test set (B), showing a considerable class imbalance but consistent distributions between data sets.


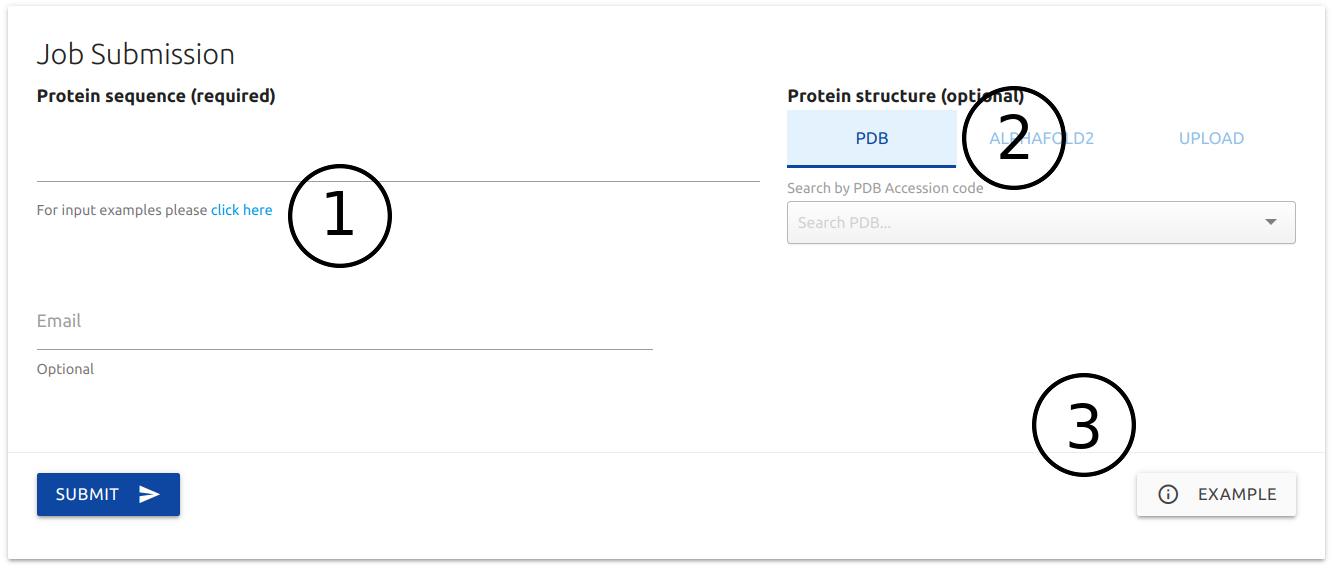


**Figure S2.** Input page for LEGO-CSM. Users are only required to provide a sequence of amino acids using the FASTA format or a plain text sequence of amino acids (1). Examples are available to assist users when preparing their input data. Alternatively, users may also provide a PDB or AlphaFold2 structure or upload a structure in PDB format (2). If an email is provided (3), users will be notified once the job finishes.


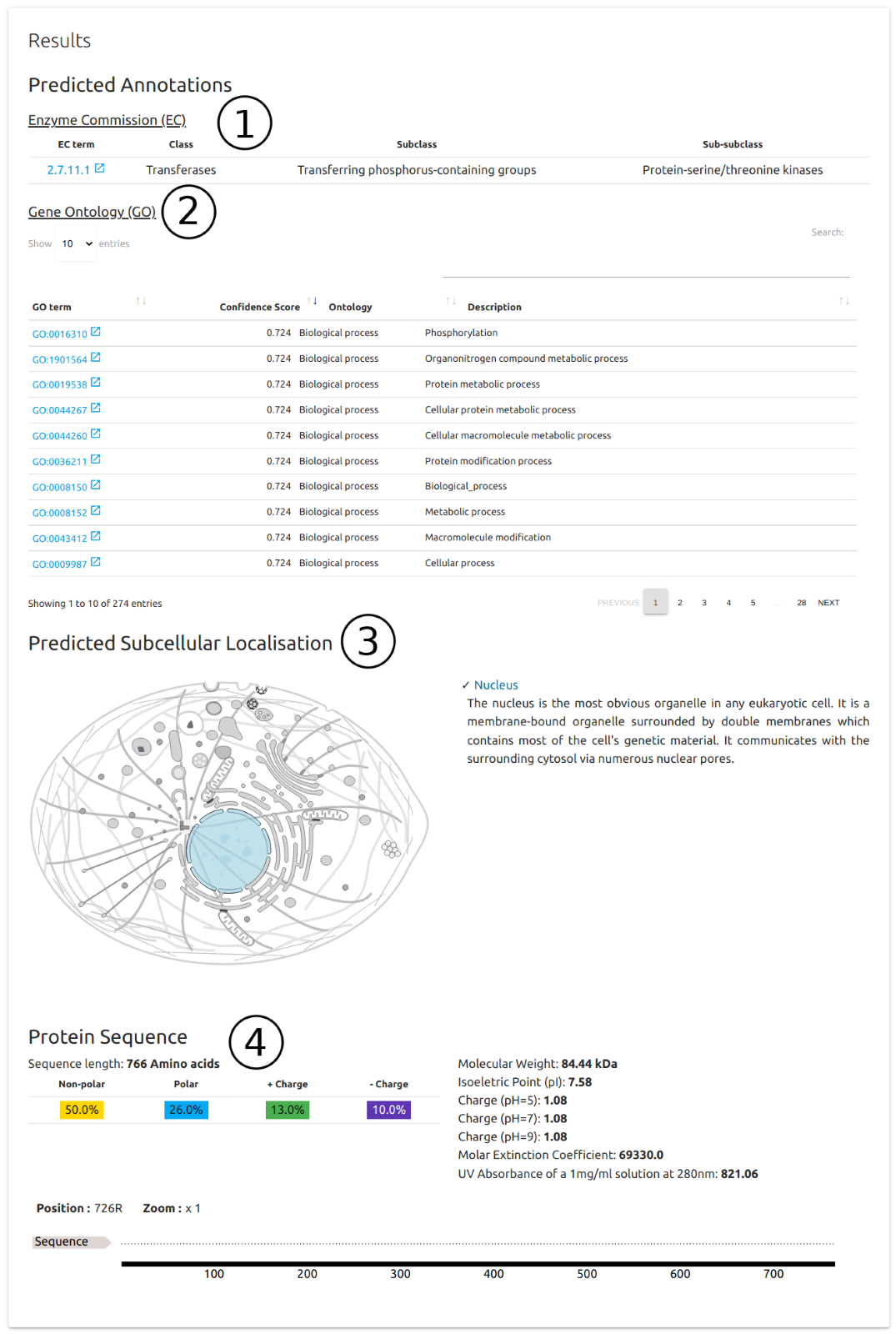


**Figure S3.** Results page for LEGO-CSM. Details on the output predictions are summarised in 4 main sections on the results page: (1) Predicted Annotations for Enzyme Commission Numbers and (2) Predicted Gene Ontology (GO) terms in table format at the top, with the classification model’s probability of that GO term being linked to the query protein being the Confidence Score; (3) Predicted Subcellular Localisation depicted as an interactive view of the cell and a brief description of the prediction; (4) and finally general details about the input protein sequence, including Isoelectric Point and UV absorbance. If a PDB structure is provided, LEGO-CSM will also display an interactive 3D with options basic options for customisation.


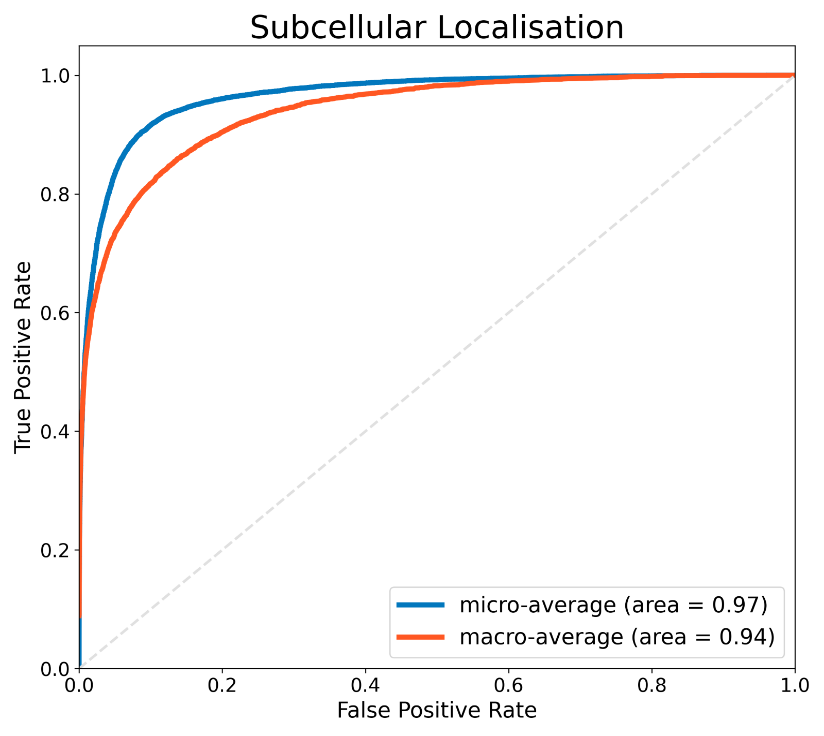


**Figure S4.** Micro- and macro-average ROC AUC plots for subcellular localisation model on 10-fold cross-validation.


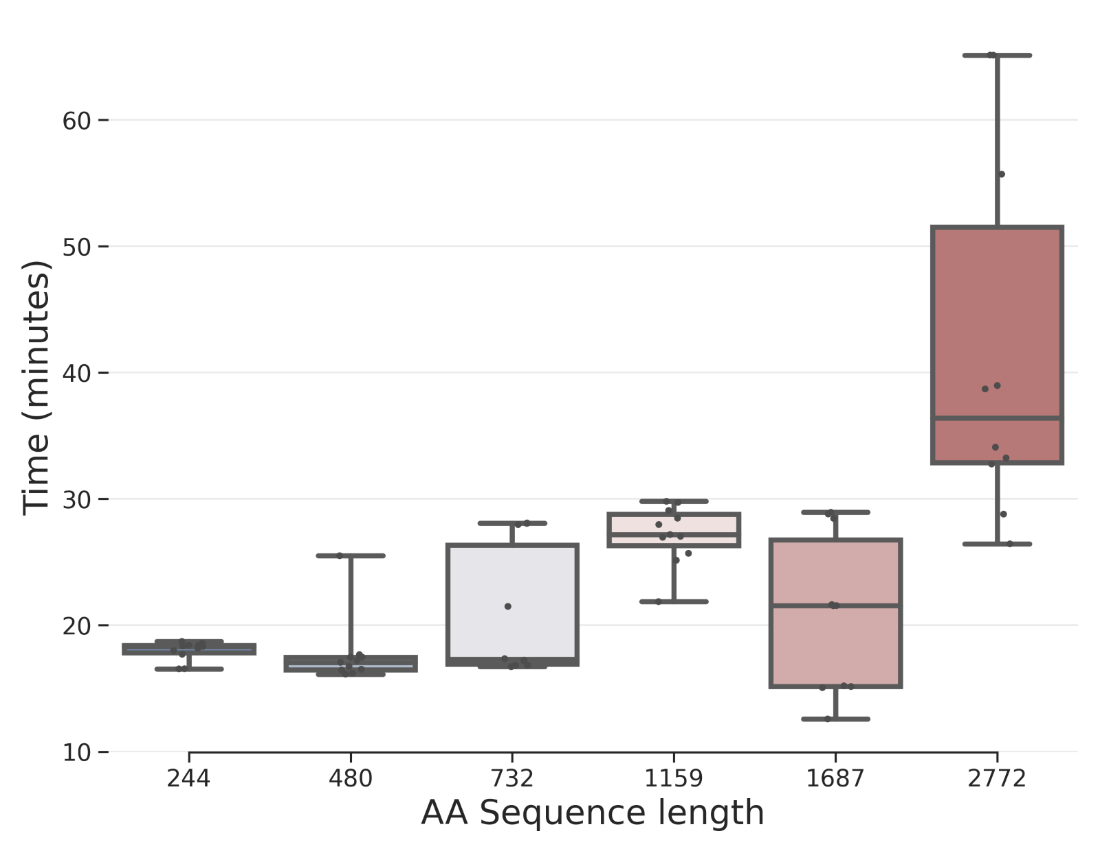


**Figure S5.** LEGO-CSM's processing time for proteins of different sizes.

**Tables**

**Table S1.** Class distribution for proteins in the EC number datasets.

| **Enzyme class name** | **Sequence-based** | | **Sequence- and structure-based** | | **#Labels** |
| --- | --- | --- | --- | --- | --- |
|  | **Train** | **Blind test** | **Train** | **Blind test** |  |
| Oxidoreductases | 18,236 | 825 | 3,116 | 212 | 173 |
| Transferases | 50,812 | 2,014 | 8,298 | 609 | 271 |
| Hydrolases | 32,381 | 1,602 | 1 | 511 | 198 |
| Lyases | 12,200 | 348 | 903 | 48 | 85 |
| Isomerases | 7,708 | 284 | 690 | 50 | 59 |
| Ligases | 15,678 | 443 | 731 | 70 | 72 |
|  | | | | | |
| Total Enzyme | 137,015 | 5,516 | 206,73 | 1,500 | 858 |
| Total Non-enzyme | 34,254 | 1,810 | 34,250 | 1,518 | 1 |
| **Total** | **171,269** | **7,326** | **54,923** | **3,018** | **859** |

**Table S2.** Class distribution for proteins in the GO terms datasets.

| **Classes** | **Sequence-based** | | | **Sequence- and structure-based** | | | **#Labels** |
| --- | --- | --- | --- | --- | --- | --- | --- |
|  | **Train** | **Blind test** | **Total** | **Train** | **Blind test** | **Total** |  |
| BP | 45,873 | 15,301 | 61,174 | 39,699 | 13,510 | 53,209 | 1,895 |
| MF | 45,250 | 10,785 | 56,035 | 38,257 | 9,268 | 47,525 | 422 |
| CC | 45,164 | 11,216 | 56,380 | 41,403 | 10,552 | 51,955 | 345 |

**Table S3.** Summary of predictive performances in terms of ROC AUC, F1-score and MCC for LEGO-CSM's main predictive components: subcellular localisation, EC numbers and GO terms.

| **LEGO-CSM Predictor** | **Data** | **Averaged**  **ROC AUC** | | **Averaged**  **F1** | | **Averaged**  **MCC** | |
| --- | --- | --- | --- | --- | --- | --- | --- |
|  |  | **CV** | **Blind** | **CV** | **Blind** | **CV** | **Blind** |
| Subcellular Localisation | Sequence | 0.929 | 0.929 | 0.546 | 0.521 | 0.466 | 0.435 |
| EC Number (Level 4) | Sequence | 0.946 | 0.930 | 0.951 | 0.908 | 0.804 | 0.730 |
|  | Sequence and Structures | 0.806 | 0.935 | 0.844 | 0.864 | 0.779 | 0.774 |
| GO Terms  (BP) | Sequence | 0.731 | 0.792 | 0.362 | 0.144 | 0.358 | 0.191 |
|  | Sequence and Structures | 0.747 | 0.786 | 0.392 | 0.142 | 0.394 | 0.189 |
| GO Terms (CC) | Sequence | 0.742 | 0.741 | 0.365 | 0.154 | 0.355 | 0.165 |
|  | Sequence and Structures | 0.748 | 0.728 | 0.384 | 0.150 | 0.374 | 0.159 |
| GO Terms (MF) | Sequence | 0.805 | 0.809 | 0.475 | 0.206 | 0.476 | 0.236 |
|  | Sequence and Structures | 0.805 | 0.807 | 0.472 | 0.204 | 0.475 | 0.232 |

**Table S4.** Comparative performance of LEGO-CSM’s subcellular localisation model with alternative approaches.

| **Method** | **Accuracy** | **Gorodkin** |
| --- | --- | --- |
| Plant-mSubP | 0.468 | 0.385 |
| MuLocDeep | 0.729 | 0.673 |
| DeepLoc | 0.736 | 0.684 |
| LEGO-CSM(CV) | 0.757 | 0.705 |
| LEGO-CSM(Blind) | 0.707 | 0.644 |

**Table S5.** Analysis of the most balanced versus imbalanced labels for subcellular localisation models.

| **Class** | **Proportion (CV)** | **Recall (10CV)** | **Proportion (Blind)** | **Recall (Blind)** |
| --- | --- | --- | --- | --- |
| Cell membrane | 0.095 | 0.584 | 0.098 | 0.586 |
| Cytoplasm | 0.194 | 0.327 | 0.183 | 0.281 |
| Endoplasmic reticulum | 0.061 | 0.251 | 0.062 | 0.237 |
| Extracellular | 0.141 | 0.921 | 0.142 | 0.883 |
| Golgi apparatus | 0.025 | 0.136 | 0.025 | 0.057 |
| Lysosome/vacuole | 0.023 | 0.047 | 0.023 | 0.047 |
| Mitochondrion | 0.108 | 0.636 | 0.109 | 0.623 |
| Nucleus | 0.288 | 0.671 | 0.291 | 0.642 |
| Peroxisome | 0.011 | 0.008 | 0.011 | 0.000 |
| Plastid | 0.054 | 0.579 | 0.055 | 0.513 |

**Table S6.** Comparative performance of LEGO-CSM on EC number prediction.

| **Level** | **ECPred** | | | | **DeepFRI**  **(Sequence-based)** | | **DeepFRI**  **(Sequence-and structure-based** | | **DeepEC** | | **Benz WS**  **(Sequence-based)** | | **LEGO-CSM**  **(Sequence-based)** | | | | **LEGO-CSM**  **(Sequence- and structure-based)** | | | |
| --- | --- | --- | --- | --- | --- | --- | --- | --- | --- | --- | --- | --- | --- | --- | --- | --- | --- | --- | --- | --- |
|  | **Training (171,269)** | | **Blind test (7,326)** | | **Blind test (7,326)** | | **Blind test (3,018)** | | **Blind test (7,326)** | | **Blind test (7,326)** | | **Training (171,269)** | | **Blind test (7,326)** | | **Training (54,923)** | | **Blind test (3,018)** | |
|  | **F1** | **MCC** | **F1** | **MCC** | **F1** | **MCC** | **F1** | **MCC** | **F1** | **MCC** | **F1** | **MCC** | **F1** | **MCC** | **F1** | **MCC** | **F1** | **MCC** | **F1** | **MCC** |
| 0 | 0.984 | 0.919 | 0.983 | 0.932 | 0.669 | 0.435 | 0.716 | 0.468 | 0.832 | 0.604 | 0.910 | 0.716 | 0.986 | 0.932 | 0.982 | 0.931 | 0.955 | 0.928 | 0.962 | 0.927 |
| 1 | 0.973 | 0.871 | 0.980 | 0.918 | 0.658 | 0.425 | 0.684 | 0.423 | 0.830 | 0.603 | 0.887 | 0.671 | 0.965 | 0.850 | 0.952 | 0.834 | 0.879 | 0.823 | 0.911 | 0.841 |
| 2 | 0.957 | 0.810 | 0.967 | 0.873 | 0.652 | 0.421 | 0.666 | 0.400 | 0.828 | 0.599 | 0.872 | 0.643 | 0.958 | 0.825 | 0.928 | 0.773 | 0.861 | 0.799 | 0.896 | 0.818 |
| 3 | 0.957 | 0.772 | 0.950 | 0.820 | 0.645 | 0.415 | 0.650 | 0.380 | 0.825 | 0.595 | 0.858 | 0.618 | 0.955 | 0.817 | 0.923 | 0.762 | 0.854 | 0.791 | 0.885 | 0.804 |
| 4 | 0.867 | 0.589 | 0.839 | 0.589 | 0.183 | 0.136 | 0.266 | -0.034 | 0.796 | 0.558 | 0.790 | 0.521 | 0.951 | 0.804 | 0.908 | 0.730 | 0.844 | 0.779 | 0.864 | 0.774 |

**Table S7.** Sequence-based performances in terms of balanced accuracy (BACC), area under the ROC curve (ROC AUC), weighted F1-score (F1W), F1-score (F1) and Matthew’s correlation coefficient across 5-fold cross-validation on the training set (5CV) and independent blind test set (Blind) for LEGO-CSM EC model at level 1.

| **Class** | **ROC AUC** | | **F1W** | | **F1** | | **MCC** | |
| --- | --- | --- | --- | --- | --- | --- | --- | --- |
|  | **5CV** | **Blind** | **5CV** | **Blind** | **5CV** | **Blind** | **5CV** | **Blind** |
| Oxidoreductases | 0.988 | 0.987 | 0.996 | 0.996 | 0.982 | 0.983 | 0.979 | 0.981 |
| Transferases | 0.987 | 0.985 | 0.991 | 0.989 | 0.984 | 0.980 | 0.977 | 0.972 |
| Hydrolases | 0.980 | 0.972 | 0.989 | 0.984 | 0.972 | 0.964 | 0.965 | 0.954 |
| Lyases | 0.981 | 0.975 | 0.997 | 0.997 | 0.977 | 0.964 | 0.975 | 0.962 |
| Isomerases | 0.989 | 0.984 | 0.999 | 0.998 | 0.985 | 0.980 | 0.984 | 0.980 |
| Ligases | 0.994 | 0.976 | 0.998 | 0.997 | 0.991 | 0.973 | 0.990 | 0.972 |

**Table S8.** Sequence- and structure-based performances in terms of balanced accuracy (BACC), area under the ROC curve (ROC AUC), weighted F1-score (F1W), F1-score (F1) and Matthew’s correlation coefficient across 5-fold cross-validation on the training set (5CV) and independent blind test set (Blind) for LEGO-CSM EC model at level 1.

| **Class** | **ROC AUC** | | **F1W** | | **F1** | | **MCC** | |
| --- | --- | --- | --- | --- | --- | --- | --- | --- |
|  | **5CV** | **Blind** | **5CV** | **Blind** | **5CV** | **Blind** | **5CV** | **Blind** |
| Oxidoreductases | 0.983 | 0.983 | 0.996 | 0.996 | 0.966 | 0.974 | 0.964 | 0.972 |
| Transferases | 0.967 | 0.976 | 0.986 | 0.986 | 0.952 | 0.966 | 0.944 | 0.958 |
| Hydrolases | 0.960 | 0.959 | 0.984 | 0.982 | 0.937 | 0.945 | 0.928 | 0.934 |
| Lyases | 0.929 | 0.937 | 0.997 | 0.997 | 0.909 | 0.913 | 0.909 | 0.913 |
| Isomerases | 0.964 | 0.960 | 0.999 | 0.998 | 0.949 | 0.948 | 0.949 | 0.948 |
| Ligases | 0.971 | 0.921 | 0.999 | 0.996 | 0.954 | 0.915 | 0.954 | 0.916 |

**Table S9.** Analysis of the most balanced versus imbalanced labels for EC models in sequence-based data. As structure-based data is a subset of sequence-based data, we have not shown it here, given similar predictive recall scores obtained.

| **Balancing Analysis** | **Label** | **Imbalance Ratio** | **Recall (10CV)** | **Recall (Blind)** |
| --- | --- | --- | --- | --- |
| Top 10 imbalanced | 1.1.1.103 | 1.37E-04 | 0.000 | 0.000 |
|  | 1.1.1.18 | 1.37E-04 | 0.000 | 0.000 |
|  | 1.1.1.205 | 1.37E-04 | 0.875 | 0.000 |
|  | 1.1.1.261 | 1.37E-04 | 0.000 | 0.000 |
|  | 1.1.1.34 | 1.37E-04 | 0.846 | 1.000 |
|  | 1.1.1.38 | 1.37E-04 | 0.000 | 0.000 |
|  | 1.1.1.8 | 1.37E-04 | 0.941 | 1.000 |
|  | 1.1.1.85 | 1.37E-04 | 0.167 | 0.000 |
|  | 1.1.1.86 | 1.37E-04 | 0.000 | 0.000 |
|  | 1.1.3.- | 1.37E-04 | 1.000 | 0.000 |
| Top 10 balanced | 2.1.1.- | 1.98E-02 | 0.919 | 0.828 |
|  | 3.1.-.- | 1.93E-02 | 0.563 | 0.724 |
|  | 2.7.11.1 | 1.81E-02 | 0.945 | 0.971 |
|  | 2.3.1.- | 1.38E-02 | 0.842 | 0.852 |
|  | 2.4.1.- | 1.37E-02 | 0.917 | 0.882 |
|  | 3.4.21.- | 9.37E-03 | 0.946 | 0.923 |
|  | 2.7.7.6 | 8.81E-03 | 0.969 | 1.000 |
|  | 2.3.2.27 | 8.67E-03 | 0.823 | 0.929 |
|  | 3.6.4.12 | 8.54E-03 | 0.872 | 0.833 |
|  | 2.5.1.- | 8.12E-03 | 0.827 | 0.933 |

**Table S10.** The average predictive performances of LEGO-CSM compared to alternative methods in terms of the area under the ROC curve (ROC AUC) on blind test sets for the three GO categories, *i.e.*, biological process (BP), cellular component (CC) and molecular function (MF).

| **GO Category** | **DeepGOPlus**  **(Sequence-based)** | **NetGO 2.0**  **(Sequence-based)** | **DeepFRI (Sequence-based)** | **DeepFRI**  **(Structure-based)** | **LEGO-CSM**  **(Sequence-based)** | **LEGO-CSM**  **(Sequence-and-structure-based)** |
| --- | --- | --- | --- | --- | --- | --- |
| BP | 0.747 | 0.607 | 0.513 | 0.528 | **0.792** | 0.786 |
| CC | 0.653 | 0.504 | 0.525 | 0.522 | **0.741** | 0.728 |
| MF | 0.699 | 0.739 | 0.588 | 0.626 | **0.809** | 0.807 |

**Table S11.** The average predictive performances of LEGO-CSM compared to alternative methods in terms of balanced accuracy (BACC) on blind test sets for the three GO categories, *i.e.*, biological process (BP), cellular component (CC) and molecular function (MF).

| **GO Category** | **DeepGOPlus**  **(Sequence-based)** | **NetGO 2.0**  **(Sequence-based)** | **DeepFRI (Sequence-based)** | **DeepFRI**  **(Structure-based)** | **LEGO-CSM**  **(Sequence-based)** | **LEGO-CSM**  **(Sequence-and-structure-based)** |
| --- | --- | --- | --- | --- | --- | --- |
| BP | 0.653 | 0.594 | 0.505 | 0.511 | **0.722** | 0.717 |
| CC | 0.652 | 0.503 | 0.510 | 0.509 | **0.655** | 0.648 |
| MF | 0.699 | **0.734** | 0.554 | 0.591 | 0.718 | 0.712 |

**Table S12.** The average predictive performances of LEGO-CSM compared to alternative methods in terms of the area under the precision-recall curve (AUPRC) on blind test sets for the three GO categories, *i.e.*, biological process (BP), cellular component (CC) and molecular function (MF).

| **GO Category** | **DeepGOPlus**  **(Sequence-based)** | **NetGO 2.0**  **(Sequence-based)** | **DeepFRI (Sequence-based)** | **DeepFRI**  **(Structure-based)** | **LEGO-CSM**  **(Sequence-based)** | **LEGO-CSM**  **(Sequence-and-structure-based)** |
| --- | --- | --- | --- | --- | --- | --- |
| BP | 0.008 | 0.234 | **0.403** | 0.354 | 0.229 | 0.226 |
| CC | 0.070 | **0.428** | 0.378 | 0.379 | 0.186 | 0.187 |
| MF | 0.048 | 0.291 | **0.307** | 0.311 | 0.238 | 0.234 |

**Table S13.** The average predictive performances of LEGO-CSM compared to alternative methods in terms of the F_max_ score on blind test sets for the three GO categories, *i.e.*, biological process (BP), cellular component (CC) and molecular function (MF).

| **GO Category** | **DeepGOPlus**  **(Sequence-based)** | **NetGO 2.0**  **(Sequence-based)** | **DeepFRI (Sequence-based)** | **DeepFRI**  **(Structure-based)** | **LEGO-CSM**  **(Sequence-based)** | **LEGO-CSM**  **(Sequence-and-structure-based)** |
| --- | --- | --- | --- | --- | --- | --- |
| BP | **0.276** | 0.157 | 0.026 | 0.040 | 0.162 | 0.163 |
| CC | **0.227** | 0.035 | 0.054 | 0.051 | 0.174 | 0.170 |
| MF | 0.296 | **0.306** | 0.126 | 0.165 | 0.227 | 0.225 |

**Table S14.** Analysis of the most balanced versus imbalanced labels for GO models using only sequence information. As structure-based data is a subset of sequence-based data, we have not shown it here given similar predictive recall scores obtained.

| **GO Category** | **Balancing Analysis** | **Label** | **Imbalance Ratio** | **Recall (10CV)** | **Recall (Blind)** |
| --- | --- | --- | --- | --- | --- |
| BP | Top 5 imbalanced | GO:1904029 | 3.27E-04 | 0.394 | 0.600 |
|  |  | GO:0007041 | 3.92E-04 | 0.289 | 0.667 |
|  |  | GO:0051588 | 3.92E-04 | 0.103 | 0.667 |
|  |  | GO:0030326 | 4.58E-04 | 0.029 | 0.143 |
|  |  | GO:0032869 | 4.58E-04 | 0.076 | 0.000 |
|  | Top 5 balanced | GO:0008150 | 4.09E-01 | 0.996 | 1.000 |
|  |  | GO:0009987 | 2.36E-01 | 0.830 | 0.986 |
|  |  | GO:0065007 | 1.62E-01 | 0.702 | 0.935 |
|  |  | GO:0050789 | 1.47E-01 | 0.702 | 0.917 |
|  |  | GO:0050794 | 1.28E-01 | 0.702 | 0.901 |
| CC | Top 5 imbalanced | GO:0000315 | 8.92E-04 | 0.533 | 0.100 |
|  |  | GO:0000324 | 8.92E-04 | 0.011 | 0.500 |
|  |  | GO:0000777 | 8.92E-04 | 0.510 | 0.600 |
|  |  | GO:0005762 | 8.92E-04 | 0.533 | 0.100 |
|  |  | GO:0005798 | 8.92E-04 | 0.108 | 0.100 |
|  | Top 5 balanced | GO:0005575 | 7.45E-01 | 0.998 | 1.000 |
|  |  | GO:0044464 | 6.25E-01 | 0.994 | 1.000 |
|  |  | GO:0044424 | 4.76E-01 | 0.916 | 0.996 |
|  |  | GO:0043226 | 3.01E-01 | 0.754 | 0.955 |
|  |  | GO:0043229 | 2.67E-01 | 0.715 | 0.914 |
| MF | Top 5 imbalanced | GO:0003725 | 9.28E-04 | 0.313 | 0.400 |
|  |  | GO:0004659 | 9.28E-04 | 0.495 | 0.500 |
|  |  | GO:0004725 | 9.28E-04 | 0.474 | 0.500 |
|  |  | GO:0005179 | 9.28E-04 | 0.532 | 0.500 |
|  |  | GO:0005253 | 9.28E-04 | 0.436 | 0.600 |
|  | Top 5 balanced | GO:0003674 | 7.79E-01 | 0.996 | 1.000 |
|  |  | GO:0005488 | 4.89E-01 | 0.880 | 0.991 |
|  |  | GO:0005515 | 3.94E-01 | 0.774 | 0.969 |
|  |  | GO:0003824 | 1.80E-01 | 0.698 | 0.890 |
|  |  | GO:0097159 | 7.79E-02 | 0.518 | 0.715 |

**References**

Almagro Armenteros, J.J.*, et al.* DeepLoc: prediction of protein subcellular localization using deep learning. *Bioinformatics* 2017;33(21):3387-3395.

Baldazzi, D.*, et al.* BENZ WS: the Bologna ENZyme Web Server for four-level EC number annotation. *Nucleic Acids Res* 2021;49(W1):W60-W66.

Carbon, S.*, et al.* AmiGO: online access to ontology and annotation data. *Bioinformatics* 2009;25(2):288-289.

Chen, Z.*, et al.* iLearnPlus: a comprehensive and automated machine-learning platform for nucleic acid and protein sequence analysis, prediction and visualization. *Nucleic Acids Res* 2021;49(10):e60.

Chen, Z.*, et al.* iFeature: a Python package and web server for features extraction and selection from protein and peptide sequences. *Bioinformatics* 2018;34(14):2499-2502.

Chen, Z.*, et al.* iLearn: an integrated platform and meta-learner for feature engineering, machine-learning analysis and modeling of DNA, RNA and protein sequence data. *Brief Bioinform* 2020;21(3):1047-1057.

da Silva, B.M.*, et al.* epitope3D: a machine learning method for conformational B-cell epitope prediction. *Brief Bioinform* 2022;23(1).

Dalkiran, A.*, et al.* ECPred: a tool for the prediction of the enzymatic functions of protein sequences based on the EC nomenclature. *BMC Bioinformatics* 2018;19(1):334.

Gligorijevic, V.*, et al.* Structure-based protein function prediction using graph convolutional networks. *Nat Commun* 2021;12(1):3168.

Hamelryck, T. and Manderick, B. PDB file parser and structure class implemented in Python. *Bioinformatics* 2003;19(17):2308-2310.

Herrera, F.*, et al.* Multilabel Classification. In: Herrera, F.*, et al.*, editors, *Multilabel Classification : Problem Analysis, Metrics and Techniques*. Cham: Springer International Publishing; 2016. p. 17-31.

Jiang, Y.*, et al.* MULocDeep: A deep-learning framework for protein subcellular and suborganellar localization prediction with residue-level interpretation. *Comput Struct Biotechnol J* 2021;19:4825-4839.

Karmakar, M.*, et al.* Analysis of a Novel pncA Mutation for Susceptibility to Pyrazinamide Therapy. *Am J Respir Crit Care Med* 2018;198(4):541-544.

Karmakar, M.*, et al.* Empirical ways to identify novel Bedaquiline resistance mutations in AtpE. *PLoS One* 2019;14(5):e0217169.

Karmakar, M.*, et al.* Structure guided prediction of Pyrazinamide resistance mutations in pncA. *Sci Rep* 2020;10(1):1875.

Kawashima, S. and Kanehisa, M. AAindex: amino acid index database. *Nucleic Acids Res* 2000;28(1):374.

Kulmanov, M. and Hoehndorf, R. DeepGOPlus: improved protein function prediction from sequence. *Bioinformatics* 2020;36(2):422-429.

Le Mercier, P.*, et al.* SwissBioPics-an interactive library of cell images for the visualization of subcellular location data. *Database (Oxford)* 2022;2022.

Mariani, V.*, et al.* lDDT: a local superposition-free score for comparing protein structures and models using distance difference tests. *Bioinformatics* 2013;29(21):2722-2728.

Meszaros, B., Erdos, G. and Dosztanyi, Z. IUPred2A: context-dependent prediction of protein disorder as a function of redox state and protein binding. *Nucleic Acids Res* 2018;46(W1):W329-W337.

Moffat, L. and Jones, D.T. Increasing the Accuracy of Single Sequence Prediction Methods Using a Deep Semi-Supervised Learning Framework. *Bioinformatics* 2021.

Myung, Y., Pires, D.E.V. and Ascher, D.B. mmCSM-AB: guiding rational antibody engineering through multiple point mutations. *Nucleic Acids Res* 2020;48(W1):W125-W131.

Myung, Y., Pires, D.E.V. and Ascher, D.B. CSM-AB: graph-based antibody-antigen binding affinity prediction and docking scoring function. *Bioinformatics* 2021.

Myung, Y.*, et al.* mCSM-AB2: guiding rational antibody design using graph-based signatures. *Bioinformatics* 2020;36(5):1453-1459.

Nguyen, T.B.*, et al.* mmCSM-NA: accurately predicting effects of single and multiple mutations on protein-nucleic acid binding affinity. *NAR Genom Bioinform* 2021;3(4):lqab109.

Nguyen, T.B., Pires, D.E.V. and Ascher, D.B. CSM-carbohydrate: protein-carbohydrate binding affinity prediction and docking scoring function. *Brief Bioinform* 2022;23(1).

Pedregosa, F.*, et al.* Scikit-learn: Machine Learning in Python. *J. Mach. Learn. Res.* 2011;12(null):2825–2830.

Pires, D.E. and Ascher, D.B. CSM-lig: a web server for assessing and comparing protein-small molecule affinities. *Nucleic Acids Res* 2016;44(W1):W557-561.

Pires, D.E. and Ascher, D.B. mCSM-AB: a web server for predicting antibody-antigen affinity changes upon mutation with graph-based signatures. *Nucleic Acids Res* 2016;44(W1):W469-473.

Pires, D.E., Ascher, D.B. and Blundell, T.L. mCSM: predicting the effects of mutations in proteins using graph-based signatures. *Bioinformatics* 2014;30(3):335-342.

Pires, D.E., Blundell, T.L. and Ascher, D.B. mCSM-lig: quantifying the effects of mutations on protein-small molecule affinity in genetic disease and emergence of drug resistance. *Sci Rep* 2016;6:29575.

Pires, D.E.*, et al.* Cutoff Scanning Matrix (CSM): structural classification and function prediction by protein inter-residue distance patterns. *BMC Genomics* 2011;12 Suppl 4:S12.

Pires, D.E.V. and Ascher, D.B. mCSM-NA: predicting the effects of mutations on protein-nucleic acids interactions. *Nucleic Acids Res* 2017;45(W1):W241-W246.

Pires, D.E.V., Rodrigues, C.H.M. and Ascher, D.B. mCSM-membrane: predicting the effects of mutations on transmembrane proteins. *Nucleic Acids Res* 2020;48(W1):W147-W153.

Portelli, S.*, et al.* Prediction of rifampicin resistance beyond the RRDR using structure-based machine learning approaches. *Sci Rep* 2020;10(1):18120.

Portelli, S.*, et al.* Understanding molecular consequences of putative drug resistant mutations in Mycobacterium tuberculosis. *Sci Rep* 2018;8(1):15356.

Rodrigues, C.H., Pires, D.E. and Ascher, D.B. DynaMut: predicting the impact of mutations on protein conformation, flexibility and stability. *Nucleic Acids Res* 2018;46(W1):W350-W355.

Rodrigues, C.H.M.*, et al.* mCSM-PPI2: predicting the effects of mutations on protein-protein interactions. *Nucleic Acids Res* 2019;47(W1):W338-W344.

Rodrigues, C.H.M., Pires, D.E.V. and Ascher, D.B. DynaMut2: Assessing changes in stability and flexibility upon single and multiple point missense mutations. *Protein Sci* 2021;30(1):60-69.

Rodrigues, C.H.M., Pires, D.E.V. and Ascher, D.B. mmCSM-PPI: predicting the effects of multiple point mutations on protein-protein interactions. *Nucleic Acids Res* 2021;49(W1):W417-W424.

Rose, A.S.*, et al.* NGL viewer: web-based molecular graphics for large complexes. *Bioinformatics* 2018;34(21):3755-3758.

Ryu, J.Y., Kim, H.U. and Lee, S.Y. Deep learning enables high-quality and high-throughput prediction of enzyme commission numbers. *Proc Natl Acad Sci U S A* 2019;116(28):13996-14001.

Sahu, S.S., Loaiza, C.D. and Kaundal, R. Plant-mSubP: a computational framework for the prediction of single- and multi-target protein subcellular localization using integrated machine-learning approaches. *AoB Plants* 2020;12(3):plz068.

Schomburg, I.*, et al.* The BRENDA enzyme information system-From a database to an expert system. *J Biotechnol* 2017;261:194-206.

Sureyya Rifaioglu, A.*, et al.* DEEPred: Automated Protein Function Prediction with Multi-task Feed-forward Deep Neural Networks. *Sci Rep* 2019;9(1):7344.

Vedithi, S.C.*, et al.* Structural Implications of Mutations Conferring Rifampin Resistance in Mycobacterium leprae. *Sci Rep* 2018;8(1):5016.

Vedithi, S.C.*, et al.* HARP: a database of structural impacts of systematic missense mutations in drug targets of Mycobacterium leprae. *Comput Struct Biotechnol J* 2020;18:3692-3704.

Wolpert, D.H. Stacked generalization. *Neural Networks* 1992;5(2):241-259.

Yao, S.*, et al.* NetGO 2.0: improving large-scale protein function prediction with massive sequence, text, domain, family and network information. *Nucleic Acids Res* 2021;49(W1):W469-W475.

Yu, C.S.*, et al.* Prediction of protein subcellular localization. *Proteins* 2006;64(3):643-651.

Zhang, M. and Zhou, Z. A Review on Multi-Label Learning Algorithms. *IEEE Transactions on Knowledge and Data Engineering* 2014;26(8):1819-1837.
